# Household food insecurity and diet diversity after the major 2010 landslide disaster in Eastern Uganda: a cross-sectional survey

**DOI:** 10.1017/S0007114515004961

**Published:** 2016-02-28

**Authors:** Peter M. Rukundo, Bård A. Andreassen, Joyce Kikafunda, Byaruhanga Rukooko, Arne Oshaug, Per Ole Iversen

**Affiliations:** 1 Department of Human Nutrition and Home Economics, Kyambogo University, Kampala, Uganda; 2 Department of Nutrition, Institute of Basic Medical Sciences, University of Oslo, PO Box 1046 Blindern, 0317 Oslo, Norway; 3 Norwegian Centre for Human Rights, Faculty of Law, University of Oslo, 0130 Oslo, Norway; 4 School of Food Technology, Nutrition and Bioengineering, Makerere University, PO Box 7062, Kampala, Uganda; 5 School of Liberal and Performing Arts, Makerere University, PO Box 7062, Kampala, Uganda; 6 Faculty of Applied Health Sciences, Oslo and Akershus University College of Applied Sciences, 0130 Oslo, Norway; 7 Department of Haematology, Oslo University Hospital, 4950 Oslo, Norway

**Keywords:** Diet diversity, Household food insecurity, Landslides, Uganda

## Abstract

In 2010, a landslide in Bududa, Eastern Uganda, killed about 350 people and nearly 1000 affected households were resettled in Kiryandongo, Western Uganda. A cross-sectional survey assessed household food insecurity and diet diversity among 1078 affected and controls. In Bududa, the affected had a lower adjusted mean score of food insecurity than controls – 9·2 (se 0·4) *v.* 12·3 (se 0·4) (*P*<0·01) – but higher diet diversity score (DDS) – 7·1 (se 0·1) *v.* 5·9 (se 0·1) (*P*<0·01). On controlling for disaster and covariates, recipients of relief food had higher food insecurity – 12·0 (se 0·6) *v.* 10·4 (se 0·3) (*P*=0·02) – whereas farmers had higher DDS – 6·6 (se 0·2) *v.* 5·6 (se 0·3) (*P*<0·01). Household size increased the likelihood of food insecurity (OR 1·15; 95 % CI 1·00, 1·32; *P*<0·05) but reduced DDS (OR 0·93; 95 % CI 0·87, <1·00; *P*=0·04). Low DDS was more likely in disaster affected (OR 4·22; 95 % CI 2·65, 6·72; *P*<0·01) and farmers (OR 2·52; 95 % CI 1·37, 4·64; *P*<0·01). In Kiryandongo, affected households had higher food insecurity – 12·3 (se 0·8) *v.* 2·6 (se 0·8) (*P*<0·01) – but lower DDS – 5·8 (se 0·3) *v.* 7·0 (se 0·3) (*P*=0·02). The latter reduced with increased age (OR 0·99; 95 % CI 0·97, 1·00; *P*<0·05), lowest education (OR 0·54; 95 % CI 0·31, 0·93; *P*=0·03), farmers (OR 0·59; 95 % CI 0·35, 0·98; *P*=0·04) and asset ownership (OR 0·56; 95 % CI 0·39, 0·81; *P*<0·01). Addressing social protection could mitigate food insecurity.

Natural disasters such as landslides, floods, earthquakes and tsunamis are now a common occurrence and often pose both acute and long-lasting challenges to humanity^(^ ^1^ ^)^. These phenomena affect households’ welfare through destruction of physical and human capital^(^ ^2^ ^)^, thus exacerbating vulnerability to food insecurity and under-nutrition. This increasing global problem has had far-reaching consequences on public health nutrition over the past few decades^(^ ^3^ ^,^ ^4^ ^)^, including suggestions for interventions to improve maternal and child nutrition during disaster and emergency situations^(^ ^5^ ^)^.

Uganda is cited among the countries on track to attain the Millennium Development Goal number one on halving the proportion of people who suffer from hunger and extreme poverty between 1990 and 2015^(^ ^6^ ^)^. However, recurrent natural disasters and related shocks affect an estimated 200 000 Ugandans annually^(^ ^7^ ^)^. Disasters are also recognised as an impediment to overall national development^(^ ^8^ ^)^ and to the implementation of the national plan for scaling-up nutrition investments^(^ ^9^ ^)^. In effect, natural disasters can further escalate the problem of under-nutrition, which has already shown an increase from an estimated five million in 1990–1992 to eleven million in 2011–2013^(^ ^10^ ^)^, most likely in tandem with an annual population growth rate of over 3 % in most districts of the country^(^ ^11^ ^)^.

Despite the consensus on the description of food security with emphasis on the availability and access at all times by everyone to safe, sufficient and nutritious food^(^ ^12^ ^)^, the indicators for measuring food insecurity vary and there is no universal gold standard to this problem^(^ ^13^ ^)^. In addition, although advances in the elaboration of the human right to adequate food yielded a United Nations General Comment Number 12 defining the right and its content^(^ ^14^ ^)^, and subsequently Voluntary Guidelines for member countries^(^ ^15^ ^)^, adapting the indicators is an enduring challenge in many countries. However, in the context of household food insecurity and diet diversity, cross-sectional designs have been helpful in establishing proxy indicators. Moreover, a number of experience-based indicators have been validated based on the access dimension of food insecurity and diet diversity, and these indicators are now recommended for developing countries where food insecurity and sub-optimal diet are still a nutritional health and development concern^(^ ^16^ ^–^ ^18^ ^)^.

In spite of the large number of people affected by natural disasters in Uganda every year, little is known about the potential impact this may have on their nutritional situation in the aftermath of such disasters. In the present study, our aim was to assess factors associated with household food insecurity and diet diversity in the aftermath of the major landslide disaster that struck the Bududa district in Eastern Uganda in 2010. Our research question assumed that the landslide disaster could have exposed households to higher food insecurity and poor diet diversity. In essence, we present relevant information about the impact of natural catastrophes – in this case a major landslide in one of Uganda’s districts – on food insecurity and diet diversity of a vulnerable population. Given that some of the landslide victims were resettled over 300 km away in the Kiryandongo district in Western Uganda, where geographical, socio-cultural and climate features seemed different from those of the Bududa district, we surveyed and treated these two districts independently.

## Methods

### Study population

The study population was households in the two districts that were hosting victims of the 2010 landslide disaster. The Bududa district was chosen because it is landslide disaster-prone^(^ ^19^ ^–^ ^22^ ^)^, and in March 2010 its sub-county of Bukalasi was the site of one of the most devastating landslides in Uganda. More than 350 persons reportedly died and over 10 000 were affected^(^ ^19^ ^,^ ^23^ ^–^ ^25^ ^)^. In addition, the Kiryandongo district was selected on the basis that it hosted nearly 1000 disaster-affected households who accepted the Government’s decision to be voluntarily resettled from Bukalasi into the Mutunda sub-county of the Kiryandongo district in the aftermath of the landslide disaster.

The two districts were examined independently in our study owing to their unique socio-cultural, geographical and ecological differences. The Bududa district in particular is of a hilly terrain and is located in Eastern Uganda on the foot of the south-western slopes of the Mount Elgon Volcano^(^ ^19^ ^)^. Average precipitation of the area is above 1500 mm of rainfall/year^(^ ^19^ ^,^ ^21^ ^)^. The population is mainly Lumasaba speaking, and the national population census of 2002 enumerated over 123 000 people and a population growth rate of 3·8 %^(^ ^26^ ^)^. The district’s population estimates for 2010 and 2011 were projected at over 167 000 and 173 000 people, respectively^(^ ^27^ ^)^, whereas in 2014 estimates indicated over 211 000 people^(^ ^28^ ^)^. On the other hand, the Kiryandongo district is of a flat terrain and is located in Western Uganda, approximately 250 km north-west of Kampala city. The rainfall is bimodal with an average of 1200 mm^(^ ^29^ ^)^. Although the estimates from the national housing and population census of 2002 reported that Kiryandongo had a population of about 190 000 people^(^ ^26^ ^)^, the population has fluctuated over time mainly due to its suitability, over the years, as a resettlement area for refugees and displaced persons^(^ ^30^ ^–^ ^32^ ^)^. In 2014, estimates indicated over 268 000 people^(^ ^28^ ^)^.

In each district, the households were categorised as affected or control. The affected group comprised landslide disaster-affected households, whereas the control group comprised households from a sub-county bordering the sub-county with the disaster-affected group. In the Bududa district, the affected households were selected from the Bukalasi sub-county, where in addition to the fatalities several households, an entire trading centre and a healthcare facility were buried by the disaster^(^ ^19^ ^,^ ^21^ ^,^ ^24^ ^)^. The control households were selected from the Bubiita sub-county – one of the neighbouring sub-counties of the affected sub-county of Bukalasi. In the Kiryandongo district, the affected households were selected from the resettled landslide disaster victims in the Mutunda sub-county, whereas the control households were selected from a randomly selected Kiryandongo sub-county (it shares the same name as the district), a neighbouring sub-county of the Mutunda sub-county.

### Selection of households and study design

In computing the household sample size, we assumed that the 19 % national estimate of under-nourishment reported in the *Uganda Nutrition Action Plan 2011–2016* was relevant for the control groups^(^ ^9^ ^)^. Owing to the absence of reliable effect measures of landslides on food insecurity and diet, we used the prevalence of under-nourishment – a state of prolonged inability to acquire enough food^(^ ^33^ ^)^ – as a proxy and assumed that the landslides had increased it by 10 % – that is, to 29 % in the affected groups. Using an equal ratio of affected:control groups, computation was made for a two-sided test based on a significance level of 5 % and power of 80 % to yield a total sample size of 576 households/district. On the basis of probability proportion to size precisions used in two recent surveys by the Uganda Bureau of Statistics^(^ ^27^ ^,^ ^34^ ^)^, we randomly targeted twelve households in a village – the smallest grouping of households from a defined enumeration area. In Uganda, a village comprises a collection of households at the lowest administrative level of a district and this is equivalent to an enumeration area during surveys^(^ ^27^ ^)^. As adopted by Uganda’s Bureau of Statistics^(^ ^27^ ^,^ ^34^ ^)^, and Harvey *et al*.^(^ ^35^ ^)^, an extra twelve households was added to each group in each district to compensate for possible non-response. We therefore targeted 300 randomly selected households/sub-county with affected households or controls – that is, a total of 600 households/district – and thus a total of 1200 households for inclusion into the study.

**Fig. 1:**
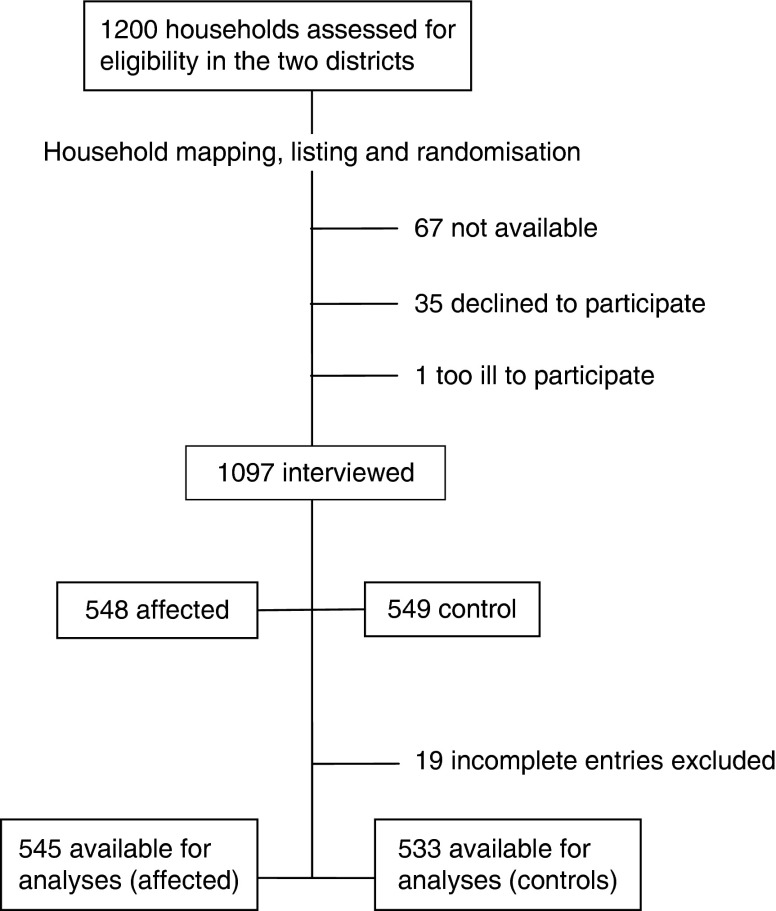
Inclusion process of the study.

Given the community organisation and the geographical localisation of the study areas, a three-stage cross-sectional survey was designed in each district. The first stage commenced with a random selection of the control sub-county from a list of sub-counties neighbouring the already known sub-county with affected households – that is, Bukalasi in the Bududa district and Mutunda in the Kiryandongo district. The assumption was that the households’ conditions of affected and controls in each district were relatively similar before the 2010 landslide disaster and subsequent events that followed. At the second stage, all the villages and their corresponding estimates of number of households in each of the affected and control areas were listed and randomly assigned into twenty-five village units using probability proportion to size, and thus giving rise to a total of 100 villages in both districts. The third stage involved randomly selecting twelve households in each village from the household lists that were generated during the pre-survey mapping and listing exercise. We used simple computer-generated random tables to obtain random numbers from a range of an ascending numbered list of village households. Households whose position on the list matched with the random numbers were identified as the index households and consulted. Fig. 1 shows the inclusion process of the study.

Ethics approval was obtained from the Uganda National Council of Science and Technology; reference number SS 2885 of 2012. The pre-survey site familiarisation visits, sensitisation meetings with districts authorities, recruitment of data collection assistants and the survey pre-test were held between 12 August and 15 November 2012. Subsequently, the data collection survey was performed from 19 November 2012 to 21 December 2012 to avoid possible bias during the Christmas and New Year festivities, given that most households often alter their usual dietary habits. Post-survey data inspection and audit commenced in January 2013, and the survey sites were closed in February 2013 after data aggregation and copy duplication were completed. Confidentiality, written consent and the other standards set by the Helsinki declaration were upheld.

### Data collection and analysis

We carried out structured interviews with heads of the households and observed relevant household features. Although we preferred to interview women respondents because of their important role in food security and nutrition, the available head of the household during the time of interviews was the one consistently consulted. Heads of households who were not available at the first visit were re-approached the following day. However, those who were not available on three visits and those not willing to be interviewed were considered as belonging to non-response households.

The main data collection tool was a questionnaire that was structured with mainly closed-ended questions. Its content included questions related to demographic and socio-economic information, experiences on access to food and the frequency and diversity of food groups consumed. Food insecurity data were assessed based on the frequency of occurrence of specific experiences within the households regarding access to food and the situation of hunger during the 30 d before the interview. We adapted questions from two previously validated and complementary tools: the Household Food Insecurity Access Scale (HFIAS) index^(^ ^17^ ^)^ and the Community Childhood Hunger Identification Project (CCHIP) index^(^ ^36^ ^,^ ^37^ ^)^. The rationale was premised on the fact that the CCHIP provides further understanding of the effects of food insecurity on household members by accounting for child hunger^(^ ^36^ ^)^. It also has a similar scoring to HFIAS, and thus provides a complementary measure to understand the food insecurity problem in resource-limited settings, especially in rural areas that rely heavily on subsistence farming and communal-based networks in times of food shortages^(^ ^38^ ^,^ ^39^ ^)^. The situation can even be more complex in situations of natural calamities such as landslide disasters, which often deprive households of their land, livelihood structures and in some cases breadwinners.

Following the questionnaire pre-test exercise, eleven experience-based indicators were adapted to obtain household food insecurity scores over a previous 30-d period. They included the following: having skipped a day without a general household meal of breakfast, lunch or supper; children ever went to bed hungry because of lack of food; children were allowed to roam and eat elsewhere because of lack of food; sought financial support to buy food; children having eaten less food because of there not being enough food; sought food assistance from neighbours, relatives and friends; limited portion sizes at meals because of there not being enough food; reduced food for adults because of there not being enough food; parents eating less because of there not being enough food; purchased food on credit; and relied on less-preferred, less-expensive food.

A food insecurity score between 0 and 3 was determined based on the frequency of occurrence of a particular experience in the last 30 d as follows: ‘never’ was scored as 0; a frequency of one to two times was considered as ‘rare’ and scored 1 point; three to ten times was considered as ‘sometimes’ and scored 2 points; and more than ten times was considered as ‘often’ and scored 3 points^(^ ^17^ ^,^ ^18^ ^,^ ^36^ ^)^. As such, a maximum score of 33 points was given if the household often reported ‘yes’ to all the eleven questions, and this was indicative of a high level of food insecurity; a total score above 0 was considered as food insecure. The higher the score meant the more food insecurity had been experienced.

Information on diet diversity was based on a retrospective recall by the head of the household about the frequency of the household eating named food items listed in an adapted semi-quantitative FFQ, similar to what had been used in the context of HIV/AIDS^(^ ^40^ ^,^ ^41^ ^)^ and in the development of *A Food Composition Table for Eastern and Central Uganda* ^(^ ^42^ ^)^. The FFQ was adapted with pre-test modifications to suit the circumstances of the survey communities. It captured food items and groups that were reported to have been eaten in the household from the day preceding the interview date and the subsequent past – that is, week and month. Commonly eaten foods (*n* 72) were listed into twelve groups: (i) cereals and grains; (ii) legumes and pulses; (iii) starchy roots and tubers; (iv) vegetables; (v) fruits and fruit juices; (vi) poultry and eggs; (vii) meat and meat products; (viii) milk and milk products; (ix) fats and oils; (x) fish and fish products; (xi) sugars and confectioneries; and (xii) condiments, spices and non-alcoholic beverages. Interviewees were asked whether the household had eaten each of the listed food items in the previous 30 d, previous 7 d and previous 24 h and the approximate frequency of use of each of the eaten items – that is, number of meals containing the food item. The information regarding food items eaten in the household over a period of 24 h preceding the interview enabled us to compute the household diet diversity score (DDS), obtained as the number of food groups eaten by the household over the 24 h before the interview. On the basis of the twelve food groups, a maximum DDS of 12 was allocated to a household that ate from all the food groups and 0 if the household members had not eaten any food at all. The DDS was used to estimate the diet quality, given its suitability in resource-limited settings^(^ ^18^ ^)^.

Potential information bias was minimised by translation of the tools from the local language back into English, pre-testing of the questionnaire before data collection and flexibility in conducting interviews in a local language in cases where the interviewee could not communicate fluently in English. In addition, a household mapping and listing exercise was carried out before household randomisation to overcome sampling bias.

### Statistical analysis

Data were analysed using SPSS statistical software version 21.0^(^ ^43^ ^)^. The GraphPad Prism version 6.0 for windows^(^ ^44^ ^)^ was also used to confirm OR and generate figures. Owing to existence of extreme values that affected normality of the data, crude mean differences in scores were tested using the Levene’s independent samples *t* test because of its suitability for application to both normally and non-normally distributed data. Given that the two dependent quantitative outcomes of food insecurity and DDS showed a moderate positive correlation (*r*<0·5 in both districts), a one-way multivariate ANCOVA (MANCOVA) model was used to test for univariate and multivariate effects while controlling for the disaster effect and socio-demographic covariates that included the following: head of the household’s sex, age and level of education, household size, main source of livelihood, existence of food security-relevant assets and having received relief food. This model was suitable, given that it also reports the adjusted univariate effect on each dependent outcome. The violation of homogeneity of variance observed with DDS posed no threat to validity, given that the Brown–Forsythe *F* and Welch’s *F* adjustments were all significant when tested in a one-way independent ANOVA before performing MANCOVA.

Subsequently, a binary logistic regression was performed to estimate the associations between exposure (disaster and socio-demographic covariates) and outcomes of food insecurity and undesirable DDS (less than six food groups). We report the likelihood to score food insecurity and undesirable diet diversity using the Wald’s test OR with corresponding 95 % CI and statistical significance of *P*<0·05. Given the ecological nature of the disaster and socio-cultural, geographical and demographic differences between the Bududa district and the Kiryandongo district, data were not pooled and the districts were treated independently in the statistical analyses.

## Results

### Characteristics of the study population

Among the 1200 eligible household representatives, 1097 household heads were interviewed, of which nineteen were excluded (Fig. 1) from the final analysis because of incompleteness. The characteristics of the study population are presented in Table 1. The mean age of the controls of the Bududa district was significantly higher compared with that of the affected households (*P*<0·01), whereas the opposite was the case in the Kiryandongo district (*P*=0·04). The mean household size was significantly lower in the affected compared with the control households of Bududa only (*P*<0·01).

**Table 1:** Characteristics of households in each district (Numbers; mean values and standard deviations)

	Bududa district (*n* 555)							Kiryandongo district (*n* 523)						
	Affected (*n* 285)			Controls (*n* 270)				Affected (*n* 260)			Controls (*n* 263)			
	*n*	Mean	sd	*n*	Mean	sd	*P*	*n*	Mean	sd	*n*	Mean	sd	*P*
(a) The interviewed head of the household														
Male	189			184			0·65	125			148			0·07
Female	96			86				135			115			
Age (years)	555	38·9	17·0		43·6	16·0	<0·01	518*	40·0	11·9		37·6	14·0	0·04
No education	50			78			<0·01	26			55			<0·01
Primary education	213			140				193			178			
Secondary education	19			46				39			25			
≥College	3			6				2			5			
(b) Household														
Number of members		5·0	3·2		6·4	3·0	<0·01		6·4	2·7		6·1	2·8	0·14
Main source of livelihood														
Farming	271			229			<0·01	225			223			0·29
Wage	1			10				6			13			
Trader	6			22				26			19			
Others	7			9				3			8			
Existence of assets that complement food source (commercial farmland, buildings, machines, motor vehicle, motorcycle, bicycle, livestock or poultry)														
Yes	93			17			<0·01	143			84			<0·01
No	192			253				117			179			
Received relief food in the last 3 years preceding the interview														
Yes	65			27			<0·01	242			4			<0·01
No	220			243				18			259			
Food insecurity scores		9·1	6·0		12·4	6·0	<0·01		9·2	8·3		5·7	5·4	<0·01
Diet diversity scores		7·1	1·9		5·9	2·3	<0·01		6·7	2·6		6·1	2·3	0·01

* There are five missing values for age in the district, four in the controls and one in the affected group.

There was a significant difference between the affected and controls in the education level of the interviewed heads of the household in both the Bududa and the Kiryandongo districts (*P*<0·01 in both). The controls in both districts had more numbers of those who had not attained any education and those with college-level education and beyond. Although a majority of the respondents had completed an education level equivalent to primary school only, the affected households in both districts had a higher number of persons who completed that level compared with their control counterparts (Table 1).

Farming was the main source of livelihood for households in both districts. However, a significant difference in the main source of livelihood was noted between the affected and the control households in the Bududa district, with a higher proportion of affected households having been involved in farming than controls. On the contrary, a lower proportion of affected than control households were involved in non-farming activities such as wage employment, trading and other activities as their main source of livelihood in that district.

Other differences in characteristics between the affected and control households in both districts were noted with respect to ownerships of food security-relevant assets and having received relief food in the past 3 years preceding the interview. Apparently, despite their perceived state of vulnerability, a higher proportion of affected households than controls in both districts reported owning some asset that complemented their food source. In addition, a higher proportion of affected households in both districts had received relief food assistance in the past 3 years – that is, from March 2010 when the landslide disaster happened in Bududa district to November–December 2012 when the survey was undertaken.

### Household food insecurity

The overall mean values and standard deviation scores for household food insecurity varied in the two districts: the affected households in the Bududa district exhibited significantly lower scores than their control counterparts, whereas in the Kiryandongo district it was the control households that exhibited significantly lower scores (Table 1). Furthermore, a similar pattern of crude differences was observed with most of the disaggregated variables (Table 2).

**Table 2:** Crude differences in food insecurity and diet diversity scores between affected and control households in each district (Numbers; mean values and standard deviations)

	Bududa district (*n* 555)						Kiryandongo district (*n* 523)					
		Affected (*n* 285)		Controls (*n* 270)				Affected (*n* 260)		Controls (*n* 263)		
	*n*	Mean	sd	Mean	sd	*P*	*n*	Mean	sd	Mean	sd	*P*
(a) Household food insecurity scores												
Sex of the interviewed head of the household												
Male	373	9·4	6·3	12·3	6·0	<0·01	273	9·9	8·4	6·1	5·2	<0·01
Female	182	8·4	5·4	12·7	6·0	<0·01	250	8·5	8·2	5·2	5·7	<0·01
Education level of the head of the household												
Primary school and less	481	8·9	6·0	12·6	5·9	<0·01	452	8·9	8·4	5·5	5·4	<0·01
Beyond primary school	74	10·5	5·9	11·8	6·4	0·43	71	11·1	7·5	7·6	5·8	0·04
Main source of livelihood												
Farming	500	9·1	5·9	12·7	5·7	<0·01	448	8·8	8·1	5·7	5·5	<0·01
Others	55	8·9	7·8	10·7	7·0	0·41	75	12·2	9·4	5·7	5·3	<0·01
Existence of assets that complement food source												
Yes	110	9·8	6·0	13·5	6·7	0·02	227	9·3	8·1	6·0	5·8	<0·01
No	445	8·7	6·0	12·4	5·9	<0·01	296	9·1	8·6	5·6	5·4	<0·01
Received relief food in the last 3 years preceding the interview												
Yes	92	11·0	5·7	14·2	5·8	0·02	246	8·7	8·0	4·0	2·7	0·03
No	463	8·5	6·0	12·2	6·0	<0·01	277	15·9	10·0	5·7	5·5	<0·01
(b) Household diet diversity scores												
The interviewed head of the household												
Male	373	7·1	2·0	6·1	2·3	<0·01	273	7·0	2·5	6·0	2·4	<0·01
Female	182	7·3	1·8	5·4	2·3	<0·01	250	6·5	2·6	6·3	2·3	0·51
Education level of the head of the household												
Primary school and less	481	7·1	1·9	5·8	2·2	<0·01	452	6·5	2·5	6·0	2·3	0·03
Beyond primary school	74	7·5	2·6	6·0	2·5	0·03	71	7·7	2·7	6·8	2·7	0·18
Main source of livelihood												
Farming	500	7·2	1·8	6·0	2·2	<0·01	448	6·7	2·6	6·0	2·3	0·01
Others	55	6·5	3·1	5·0	2·4	0·07	75	7·2	2·3	6·7	2·5	0·34
Existence of assets that complement food source												
Yes	110	7·1	1·9	6·5	1·8	0·26	227	7·3	2·5	6·3	2·2	<0·01
No	445	7·2	2·0	5·8	2·3	<0·01	296	6·0	2·4	6·1	2·4	0·84
Received relief food in the last 3 years preceding the interview												
Yes	92	6·9	2·0	6·9	1·8	0·90	246	6·9	2·5	6·5	3·1	0·77
No	463	7·2	1·9	5·8	2·3	<0·01	277	4·8	2·4	6·1	2·3	0·02

On adjusting for the socio-demographic covariates (Table 3), the multivariate analysis model showed that in the Bududa district the control households exhibited higher mean scores of food insecurity compared with those affected: 12·3 (se 0·4) *v.* 9·2 (se 0·4) (*P*<0·01). Recipients of relief food also had higher food insecurity than those who did not receive it when the disaster and covariates were controlled: 12·0 (se 0·6) *v.* 10·4 (se 0·3) (*P*=0·02). On the contrary, in Kiryandongo, affected households had higher food insecurity than controls – 12·3 (se 0·8) *v.* 2·6 (se 0·8) (*P*<0·01) – whereas the recipients of relief food experienced less food insecurity than their counterparts who did not receive it – 3·9 (se 0·9) *v.* 10·7 (se 0·8) (*P*<0·01).

**Table 3:** Adjusted differences in household food insecurity and diet diversity scores (Numbers; mean values with their standard errors)

	Bududa district								Kiryandongo district							
		ANCOVA*								ANCOVA*						
		Food insecurity†			Diet diversity‡			MANCOVA§		Food insecurity†			Diet diversity‡			MANCOVA§
Variables	*n*	Mean	se	*P*	Mean	se	*P*	*P*	*n*	Mean	se	*P*	Mean	se	*P*	*P*
Disaster																
Affected	285	9·2	0·4	<0·01	7·1	0·1	<0·01	<0·01	259	12·3	0·8	<0·01	5·7	0·3	0·01	<0·01
Controls	270	12·3	0·4		5·9	0·1			259	2·6	0·8		7·2	0·3		
Sex																
Male	373	10·9	0·3	0·25	6·6	0·1	0·25	0·27	270	7·9	0·4	0·10	6·5	0·2	0·66	0·22
Female	182	10·3	0·4		6·4	0·2			248	6·9	0·4		6·4	0·2		
Education																
Above primary school	74	10·5	0·7	0·79	6·8	0·3	0·33	0·59	71	9·0	0·8	0·06	7·1	0·3	0·02	0·01
Primary school and less	481	10·7	0·3		6·5	0·1			447	7·2	0·3		6·3	0·1		
Main livelihood																
Farming	500	10·9	0·3	>0·05	6·6	0·1	<0·01	<0·01	443	7·3	0·3	0·14	6·4	0·1	0·22	0·13
Others	55	9·3	0·8		5·6	0·3			75	8·6	0·8		6·7	0·3		
Had asset to complement food source																
Yes	110	10·9	0·6	0·72	6·5	0·2	0·95	0·94	225	7·4	0·5	0·81	6·9	0·2	<0·01	<0·01
No	445	10·7	0·3		6·5	0·1			293	7·5	0·4		6·1	0·1		
Having received relief food																
Yes	92	12·0	0·6	0·02	6·6	0·2	0·74	0·06	245	3·9	0·9	<0·01	7·4	0·3	<0·01	<0·01
No	463	10·4	0·3		6·5	0·1			273	10·7	0·8		5·6	0·3		

MANCOVA, multivariate ANCOVA.

* Test for the univariate effect of each variable on the outcome after adjusting for covariates.

† Covariates in the model included whether a household was affected by the disaster, head of the household’s sex, age and education attained, household size, main source of livelihood, existence of assets to complement food source, whether the household had received relief food and diet diversity.

‡ Covariates in the model included whether a household was affected by the disaster, head of the household’s sex, age and education attained, household size, main source of livelihood, existence of assets to complement food source, whether the household had received relief food and food insecurity.

§ Test for multivariate effect of each variable on both outcomes after adjusting for covariates. Given two dependent variables in the model, Hotelling’s Trace value is reported.

The binary logistic regression model showed that only household size seemed to predict food insecurity in Bududa district when the disaster and socio-demographic covariates were taken into account (Table 4): an increase in household size exhibited a higher likelihood for a household to experience food insecurity (OR 1·15; 95 % CI 1·00, 1·32; *P*<0·05). In Kiryandongo, the disaster and sex of the household head predicted food insecurity (Table 4); although affected households had higher food insecurity scores than controls, they had a lower likelihood to experience food insecurity when socio-demographic variables were controlled (OR 0·24; 95 % CI 0·08, 0·95; *P*=0·04). On the other hand, female-headed households were nearly twice more likely to experience food insecurity compared with their male counterparts (OR 1·56; 95 % CI 1·01, 2·42; *P*<0·05).

**Table 4:** Binary logistic regression model predicting the households’ likelihood to experience food insecurity and undesirable diet diversity in Bududa and Kiryandongo districts (Numbers and percentages; odds ratios and 95 % confidence intervals)

	Food insecurity					Undesirable diet diversity		
Variables	*n*	%	OR	95 % CI	*P*	OR	95 % CI	*P*
(a) Bududa district								
Disaster effect								
Controls	270		1			1		
Affect	285		0·82	0·33, 2·06	0·67	4·22	2·65, 6·72	<0·01
Head of the household								
Male	373		1			1		
Female	182		0·96	0·42, 2·23	0·93	0·73	0·48, 1·10	0·14
Education of the head of the household								
Beyond primary	74		1			1		
Primary and below	481		2·23	0·48, 10·31	0·31	0·91	0·51, 1·62	0·75
Main source of livelihood								
Others	55		1			1		
Farming	500		0·37	0·l2, 1·14	0·08	2·52	1·37, 4·64	<0·01
Had a food security-relevant asset								
Yes	110		1			1		
No	445		0·54	0·20, 1·43	0·22	0·89	0·50, 1·61	0·71
Had received relief food in the last 3 years								
No	463		1			1		
Yes	92		0·16	0·02, 1·23	0·08	1·05	0·58, 1·89	0·87
Age (years)	555		0·98	0·96, 1·01	0·13	1·01	<1·00, 1·02	0·09
Household size	555		1·15	1·00, 1·32	<0·05	0·93	0·87, <1·00	0·04
Diet diversity scores	555		0·89	0·73, 1·08	0·22			
Food insecurity scores	555					1·00	0·97, 1·04	0·99
Hosmer and Lemeshow test (*P*)					0·99			0·73
Nagelkerke’s *R* ^2^	0·09	9				0·18		
Overall predictive accuracy (%)		95				72		
(b) Kiryandongo district								
Disaster effect								
Controls	259		1		0·04	1		0·23
Affect	259		0·24	0·08, 0·95		0·55	0·21, 1·47	
Head of household								
Male	270		1		<0·05	1		0·54
Female	248		1·56	1·01, 2·42		0·89	0·61, 1·29	
Education attained								
Beyond primary	71		1		0·10	1		0·03
Primary and below	447		1·93	0·87, 4·25		0·54	0·31, 0·93	
Main source of livelihood								
Others	75		1		0·34	1		0·04
Farming	443		1·39	0·71, 2·72		0·59	0·35, 0·98	
Had a food security-relevant asset								
Yes	225		1		0·50	1		<0·01
No	293		0·86	0·54, 1·35		0·56	0·39, 0·81	
Had received relief food in the last 3 years								
No	273		1		0·16	1		0·11
Yes	245		2·43	0·70, 8·42		2·19	0·84, 5·75	
Age (years)	518		0·99	0·97, 1·01	0·27	0·99	0·97, 1·00	<0·05
Household size	518		1·00	0·93, 1·09	0·92	0·94	0·88, >1·00	0·07
HFIS	518					1·00	0·98, 1·03	0·94
HDDS	518		1·05	0·96, 1·15	0·32			
Hosmer and Lemeshow test (*P*)					0·57			0·17
Nagelkerke’s *R* ^2^	0·05	5				0·10		
Overall predictive accuracy (%)	0·79	79				63		

HFIS, household food insecurity scale; HDDS, household diet diversity score.

### Household diet diversity

**Fig. 2:**
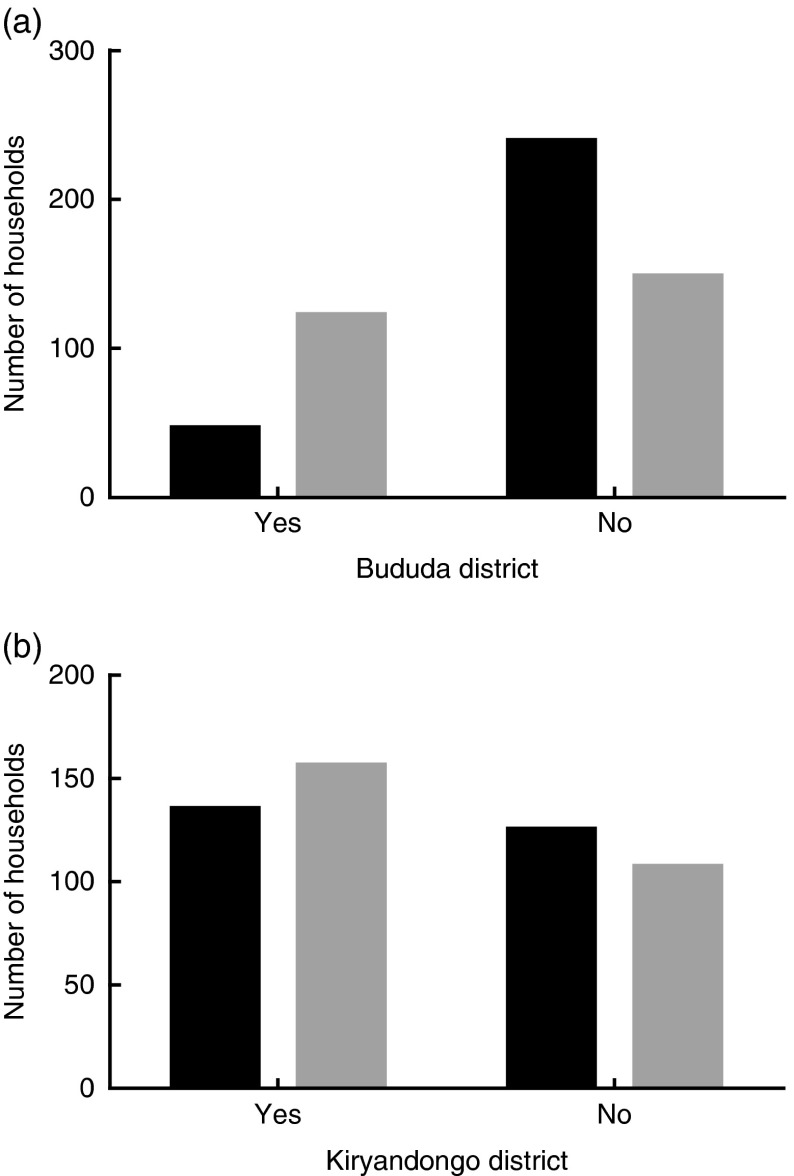
Likelihood to score undesirable diet diversity in Bududa (a) and Kiryandongo (b) districts. 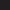, Affected; 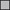, control.

Crude household DDS were significantly higher among the affected than control households in both the Bududa and the Kiryandongo districts (Table 1). Although this consistent pattern of differences was sustained on further analysis by stratification, the significance was gradually lost on some of the disaggregated variables (Table 2). Moreover, when we applied the DDS of six of the twelve food groups (50 %) and above as a cut-off for a desirable DDS to form two categorical outcomes, the affected households in the Bududa district were less likely than their control counterparts to score a low (undesirable) DDS of less than six food groups (crude OR 0·23; 95 % CI 0·16, 0·35; *P*<0·01; Fig. 2(a)). In the Kiryandongo district, the crude OR was insignificant (Fig. 2(b)).

On adjusting for socio-demographic covariates, the model showed that affected households had a higher mean for DDS in the Bududa district – 7·1 (se 0·1) *v.*5·9 (se 0·1) (*P*<0·01) – whereas households whose main source of livelihood was farming had a higher DDS than that of households with other sources of livelihood when the disaster and socio-demographic covariates were taken into account: 6·6 (se 0·1) *v.* 5·6 (se 0·3) (*P*<0·01). In Kiryandongo district, affected households had lower DDS than controls: 5·7 (se 0·3) *v.*7·2 (se 0·3) (*P*=0·01) – that is, controlling for covariates in the model favoured controls in Kiryandongo to reverse the crude difference that was previously in favour of affected households. In this district, it was also observed that households with assets that complemented food source and recipients of relief food exhibited higher DDS that those who had no such assets and those who did not receive relief food when the disaster and covariates were taken into account: 6·9 (se 0·2) *v.* 6·1 (se 0·1) (*P*<0·01) and 7·4 (se 0·3) *v.* 5·6 (se 0·3) (*P*<0·01), respectively (Table 3).

The regression model showed that the disaster, main source of livelihood and household size were significant predictors of undesirable diet diversity in Bududa district (Table 4): disaster-affected households were more than four times likely to score below six food groups than their control counterparts (OR 4·22; 95 % CI 2·65, 6·72; *P*<0·01). In addition, households whose main livelihood was farming were nearly three times more likely to score below six food groups than those whose main livelihood source was not farming (OR 2·52; 95 % CI 1·37, 4·64; *P*<0·01), whereas increase in household size seemed to lower the likelihood of households to score below six food groups (OR 0·93; 95 % CI 0·87, <1·00; *P*=0·04). In Kiryandongo, all the four significant predictors were associated with lowering the likelihood of a household to score below six food groups: primary-level education and below (OR 0·54; 95 % CI 0·31, 0·93; *P=*0·03); farming as a main source of livelihood (OR 0·59; 95 % CI 0·35, 0·98; *P*=0·04); not having an asset to complement food source (OR 0·56; 95 % CI 0·39, 0·81; *P*<0·01) and age of the head of the household (OR 0·99; 95 % CI 0·97, 1·00; *P*<0·05).

### Multivariate effects on both food insecurity and diet diversity

Given the positive correlation of the two dependent variables in the multivariate model – that is, food insecurity and diet diversity – the MANCOVA test of multivariate effect showed that the disaster, which was treated as a fixed factor in the analysis, was the only variable that predicted both outcomes when socio-demographic variables were controlled in the Bududa and Kiryandongo districts (*P*<0·01 in both) (Table 3). Distinctively, the main source of livelihood could predict both outcomes in the Bududa district only (*P*<0·01), whereas in the Kiryandongo district both outcomes could be predicted by education (*P*=0·01), owning a relevant asset that complements food source (*P*<0·01) and having received relief food (*P*<0·01). On the other hand, the Wald’s test of the logistic regression model showed that the landslide disaster had varying effects in the two districts when socio-demographic variables were controlled. In Bududa, it could only significantly predict a low DDS of less than six food groups with an overall predictive accuracy of 72 % and variance (Nagelkerke’s *R* ^2^) of 18 % (Table 4), whereas in Kiryandongo it could predict food insecurity with a predictive accuracy of 79 and 5 % variance (Table 4).

## Discussion

We have shown that disaster-affected households and their control counterparts differed significantly in various characteristics including food insecurity and diet diversity. Notably, although household food insecurity scores were higher in controls of the Bududa district, in the Kiryandongo district, it was the affected households that presented higher scores. Intriguingly, although the disaster-affected households performed better than their control counterparts on diet diversity with higher crude scores in both districts, the trend was reversed after controlling for socio-demographic covariates in Kiryandongo. On the other hand, the regression model showed that increase in household size increased the likelihood of food insecurity but reduced the likelihood of a DDS of less than six food groups in Bududa. A low DDS was also associated with whether the household was affected by the disaster and whether farming was a main source of livelihood for the household. In Kiryandongo, food insecurity was less likely in disaster-affected households, yet twice likely in female-headed households when the disaster and other socio-demographic variables were controlled. Moreover, the likelihood to have a DDS of less than six food groups was reduced when the head of the household had an education level not exceeding primary school, farming as the main source of livelihood, no asset to complement food source and a higher age.

In view of the existing national policy commitment to enhance plans to mitigate the effects of disasters on nutritional health within the context of scaling-up nutrition investments in Uganda^(^ ^9^ ^)^, our findings seem to imply that the situation of food insecurity and diet of vulnerable households affected by landslide disasters should not be considered in isolation from unaffected counterparts in the districts where disasters strike. Given that the control households in the Kiryandongo district scored the lowest on food insecurity, it may be plausible to argue that, in the context of food security, the resettled landslide-affected victims in the Kiryandongo district were facing more food insecurity challenges than their neighbours in the surroundings, but their situation was less challenging compared with those who remained in the Bududa district, including the control area neighbouring the disaster area. It might also be possible that those who were resettled in the Kiryandongo district had not yet fully recovered from the effects of deprivation and resettlement, thus the higher food insecurity scores. Moreover, an assessment of the food and nutrition security performed in the Kiryandongo district in March 2013 reported that more than one-third of assessed households in the district experienced moderate-to-severe hunger^(^ ^45^ ^)^.

Given that this study considered affected and control households residing in different sub-counties of two distinct districts, it is possible to deduce that the differences in food insecurity that were observed in the study districts could have been attributed to factors that are grounded in the socio-cultural and geographical architecture of the independent population groups that were surveyed. As noted by Vakis^(^ ^46^ ^)^, household food insecurity during disaster can also be exacerbated by often uninvestigated circumstances, which in this case may have influenced affected households on different scales in the period before, during or after the disaster. As such, the conditions and ingredients of the resettlement process of some of the landslide victims from the Bududa district into previously inhabited and distantly apart areas in the Kiryandongo district could also have influenced the outcomes observed in the affected groups.

As would be expected, a significant proportion of disaster-affected households should have received relief food assistance. However, in Bududa, most affected households reported not receiving relief food, whereas most affected households in Kiryandongo had received it. Although this seems to concur with the Government reports that extensive relief assistance was procured and distributed to the disaster victims in the aftermath of the landslide disaster^(^ ^23^ ^,^ ^24^ ^)^, it may be possible that circumstances in Kiryandongo allowed more affected households to access relief food as shown in the findings. Although this study did not investigate differences within groups to critically evaluate the effect of relief food on individual households, the potential of relief food influence on household food security could be seen in both districts despite opposite trends – that is, increased food insecurity in Bududa but reduced food security in Kiryandongo. In the latter district, recipients of relief food also had higher DDS.

Although most of these findings may not contradict previous studies that have found a positive effect of relief food on food insecurity and diet^(^ ^41^ ^,^ ^47^ ^)^, it should be noted that the data collected by this study had a different focus and design that did not place much emphasis on the quality and quantity of relief food and related dynamics. It may therefore be insufficient to explain the detailed effect of relief food in the circumstances of post-landslide disaster situations in the study areas that were surveyed. More empirical studies on the biological effect and adequacy of relief food are necessary so as to provide more robust conclusions on the extent to which relief food may offer a buffer or protective effect against food insecurity and sub-optimal diet diversity.

Although the findings in Bududa district are in harmony with other studies showing that household size increases the risk of food insecurity in vulnerability settings in Uganda^(^ ^40^ ^,^ ^47^ ^)^, its effect on DDS seems to be the reverse as increased size reduced the likelihood to score a low diversity of less than six food groups. It was also apparent in Kiryandongo district that higher DDS were observed in households with the household head having an educational level beyond primary school and with assets that complement food source. From a public health and development policy perspective, these findings seem to suggest that the negative effects of disasters on nutritional health are a complex and protracted outcome that may be mediated through the dynamic aspects in the household’s social environment, often beyond the radar of a cross-sectional investigation. Sustainable mitigation in the long term may require that deliberate efforts are instituted to amplify the integration of nutritional interventions in disaster management and other cross-cutting national programmes on socio-economic empowerment, sustained education promotion and access and asset security. It is also vital, during resettlement of affected households from disaster-prone areas, to ensure that food and nutrition-sensitive remedy and recourse measures are specified and pursued so that interventions can meet expectation and assessed needs.

Our findings do not contradict the evidence that sex plays an important role in food security, especially that women are important stakeholders in household food and nutrition security and can potentially influence household economic security for improved nutritional health^(^ ^48^ ^)^. Given the available evidence on the health consequences of maternal and child under-nutrition that has triggered a global consensus and momentum to scale-up nutrition investments targeting women of reproductive age and children^(^ ^49^ ^,^ ^50^ ^)^, we observed that women-headed households in the disaster-affected group had nearly twice the likelihood to score food insecurity in the Bududa district. Similar to previous studies that have shown that women-headed households are at a higher risk of food insecurity in Uganda^(^ ^51^ ^,^ ^52^ ^)^, our findings reinforce the arguments that advance the need to broaden the focus towards women empowerment as a means to improve household food insecurity. On the other hand, it also implies the need for balanced interventions that are sensitive to vulnerability of the male sex during situations of disaster. In effect, narrowing the disparities in food insecurity and diet diversity that may arise because of sex differences within and between households in disaster-prone areas should be among the targets of disaster management and related nutritional interventions at various levels of the State.

In this study, we did not correct for possible effects of seasonal variations on diet and food insecurity, as the cross-sectional survey was performed in the last quarter of the year. Given that both districts experience a bimodal rainfall distribution, it was expected that most farming households were involved or preparing for the second season of harvests during that period^(^ ^53^ ^)^, which is a potential bias. The ecological nature of the disaster also prevented sampling of both affected and control households from a homogeneous population. The disaster was widespread in social and geographical scope, and the subsequent resettlement was in a specific and previously unoccupied locality. It was therefore difficult to locate suitable affected and controls households from within the same population group. Moreover, because of inherent differences in a number of factors between the Bududa and Kiryandongo districts, we decided to limit our comparisons between the affected and control households within each district rather than across the two districts, as such a comparison would be flawed both from a statistical and clinical perspective. Furthermore, given the inconsistencies in scores between the two districts to the extent that the directions of some results are opposite, generalisability of findings could have been undermined beyond geographical settings and types of natural disaster. It is therefore possible that some differences between affected and control households could have arisen because of differences in socio-demographic and other factors at the district and sub-county level.

Other limitations are the absence of nationally validated and standardised FFQ and food insecurity assessment tools for use in Uganda, the lack of measures of body composition and biomarkers for nutritional intake and potential recall bias as we relied on information provided by the available head of the household. We also note, as reported in our previous findings^(^ ^54^ ^)^, an external weakness linked to capacity limitations of a loosely centralised national disaster management institutional structure that seems to be heavily reliant on a host of humanitarian actors. The set-up of the governance system makes it difficult to trace details and specification on disaster-related food and nutrition interventions in the two surveyed districts. However, the major strength of our study was in the adaptation of two complementary tools to score food insecurity while taking into account child hunger – that is, the HFIAS and CCHIP – and the systematic implementation of a multi-stage random sampling survey procedure in two districts that hosted households that had been affected by the same phenomenon, but were later separated by a voluntary government intervention of resettlement.

In view of our findings, we affirm previous observations urging the State authorities to establish mechanisms that protect households against disaster-related food insecurity shocks and exposures to sub-optimal diet during and after disasters have occurred. Affected communities should be mapped and empowered with the relevant means to acquire and effectively utilise relevant entitlements such as land for agriculture, livestock and equipment to advance their prospects against food insecurity and diet inadequacy. Furthermore, in line with the recommendations on the human right to adequate food contained in the United Nations General Comment 12 of 1999^(^ ^14^ ^)^, emergency and humanitarian interventions occasioned by the State should gradually focus on increasing the capacity of households to produce and procure their own food. In this case, it seemed apparent that a small household size, relief food interventions as well as encouraging formal education beyond primary school and alternative economic potential beyond the mere reliance on agricultural farming for food and livelihood may provide a dignified and long-term insulation and insurance against extreme disaster-related shocks.

It is necessary for the State and the constituent districts to adopt a robust social protection framework targeting vulnerable disaster-affected households. Such an approach can use scores of food insecurity and diet diversity as core proxies to inform nutrition interventions of any scale and magnitude during precarious and fragile situations of disaster. In the process, other complementary actions that may be helpful include an enabling environment to facilitate increased access to emergency relief food, capital, cash transfers, public works to facilitate economic activities, micro-credit schemes, food transfers, service fee waivers, employment guarantees and remedial compensations that include resettlement of vulnerable households among others^(^ ^46^ ^)^. Such fiscal and statutory measures, if backed by implementable policy and legislation, may provide some desired form of extended insurance against commitment of already-constrained household resources to buy food and other amenities in the aftermath of disasters. This approach may also strengthen the State’s capacity to deal with its human rights obligation to protect at-risk households against disaster-related deprivation and its effects.

Finally, the present results indicate that the factors associated with household food insecurity and diet diversity in the aftermath of a disaster are dynamic and seem to be influenced by the social construction of households. In countries such as Uganda where most households in rural areas have relatively low incomes and are faced with relatively large income inequalities and deprivation, it cannot be taken for granted that all disaster-affected households are worse-off than unaffected counterparts in terms of food insecurity and diet diversity. The situation may differ from one district to another depending on where the disaster occurred and its nature, in particular the extent of deprivation and the circumstances of each household before, during and after a disaster. Therefore, disaster management processes and interventions should position food and nutrition remedies among the critical priorities within an overarching framework that provides social protection and livelihood security, while respecting cultural and geographical diversity at the points of intervention.
